# SARS-CoV-2 surveillance in indoor and outdoor size-segregated aerosol samples

**DOI:** 10.1007/s11356-022-20237-7

**Published:** 2022-04-21

**Authors:** Álvaro del Real, Andrea Expósito, Laura Ruiz-Azcona, Miguel Santibáñez, Ignacio Fernández-Olmo

**Affiliations:** 1 Medicine and Psychiatry Department, Universidad de Cantabria, Av. Cardenal Herrera Oria, s/n, 39011 Santander, Cantabria, Spain; 2 Departamento de Ingenierías Química y Biomolecular, Universidad de Cantabria, Avda. Los Castros S/N, 39005 Santander, Cantabria, Spain; 3 Global Health Research Group. Dpto Enfermería, Universidad de Cantabria, Avda. Valdecilla, s/n, 39008 Santander, Cantabria, Spain; 4 Nursing Research Group, IDIVAL, Calle Cardenal Herrera Oria s/n, 39011 Santander, Cantabria, Spain

**Keywords:** COVID-19, SARS-CoV-2, Particulate matter (PM), Aerosol, Environmental surveillance, Low-volume air sampling

## Abstract

We aimed to determine the presence of SARS-CoV-2 RNA in indoor and outdoor size-segregated aerosol samples (PM_10-2.5_, PM_2.5_). Five outdoor daily samples were collected between November and December 2020 in an urban/industrial area with relatively high PM_10_ levels (Maliaño, Santander, Spain) by using a PM impactor (air flowrate of 30 L/min). In a non-hospital indoor sampling surveillance context, 8 samples in classrooms and 6 samples in the central library-Paraninfo of the University of Cantabria (UC) were collected between April and June 2021 by using personal PM samplers (air flowrate of 3 L/min). Lastly, 8 samples in the pediatric nasopharyngeal testing room at Liencres Hospital, 6 samples from different single occupancy rooms of positive patients, and 2 samples in clinical areas of the COVID plant of the University Hospital Marqués de Valdecilla (HUMV) were collected between January and May 2021. N1, N2 genes were used to test the presence of SARS-CoV-2 RNA by RT-qPCR. SARS-CoV-2 positive detection was only obtained from one fine fraction (PM_2.5_) sample, corresponding to one occupancy room, where a patient with positive PCR and cough was present. Negative results found in other sampling areas such as the pediatric nasopharyngeal testing rooms should be interpreted in terms of air sampling volume limitation and good ventilation.

**Supplementary Information:**

The online version contains supplementary material available at 10.1007/s11356-022-20237-7.

## Introduction

Since the occurrence of the first coronavirus disease 2019 (COVID-19) positive cases due to the severe acute respiratory syndrome coronavirus 2 (SARS-CoV-2) in Wuhan (China) and its rapid spread worldwide, important questions have arisen about the main reasons for its rapid spread and transmission among the population, which has so far resulted in more than 380 million cases and more than 5.6 million deaths worldwide (World Health Organization 2022). At the early stages of the SARS-CoV-2 pandemic, human respiratory droplets and direct contact were assumed to be as main transmission routes, being aerosol transmission poorly understood (Asadi et al. 2020; Morawska and Cao 2020; World Health Organization 2020). Increasing evidence that SARS-CoV-2 is transmitted through aerosols is nowadays more conclusive as more and more studies have been published since first supporting indoor results in Wuhan hospitals (Liu et al. 2020), extended posteriorly to other hospital and non-hospital indoor air environments (Birgand et al. 2020; Bazzazpour et al. 2021; Borges et al. 2021; Comber et al. 2021; Grimalt et al. 2021; Noorimotlagh et al. 2021).

Definition of “aerosol” varies across different publications, so briefly, aerosol is a suspension of solid particles or liquid droplets (size ≤ 100 μm) in air. Whereas large droplets (size greater than 100 μm) settle down close to the source transmissible patient, smaller aerosol particles stay aloft and can drift long distances. Once inhaled, the smallest particles can reach deeper into the pulmonary region. Larger particles remain in the nasopharyngeal region, whereas intermediate-sized particles are captured in the tracheobronchial region of the upper respiratory system (Pan et al. 2019; Milton 2020). A recent report in The Lancet (Greenhalgh et al. 2021) highlights the ten most important reasons to strengthen the aerosol transmission route of SARS-CoV-2. The authors of the report emphasize how difficult it is to prove transmission by this route, but point out that there are many more arguments in favor than against it. One of the main arguments is that certain infections cannot be explained by other routes such as droplets and fomites. They also point to the need for evidence of the viable virus in aerosols.

In relation to the outdoor transmission of SARS-CoV-2 through aerosols, few published studies exist (Setti et al. 2020a; Kayalar et al. 2021; Linillos-Pradillo et al. 2021) with significant gaps in the importance of these specific outdoor transmission pathways (Bulfone et al. 2021). In this context, the possibility that air pollution may have implications for SARS-CoV2 transmission, in particular through particulate matter (PM), was considered (Santurtún et al. 2022). In this regard, the “Italian Society of Environmental Medicine (SIMA)” hypothesized that PM could play a role in the spread of SARS-CoV-2 in the most affected regions of Italy at the early stages of the COVID-19 pandemic (SIMA 2020), being evidence of its presence even published in PM_10_ samples (particles with aerodynamic diameter < 10 μm) from northern Italy (Setti et al. 2020a) in line with the evidence already available for other viruses (Zhao et al. 2019), suggesting the presence of SARS-CoV-2 on PM_10_ as an early epidemic indicator of recurrence (Setti et al. 2020b). In addition, detection of SARS-CoV-2 in other environmental matrices, such as wastewater together with airborne detection, seems to be useful in environmental SARS-CoV-2 surveillance and risk monitoring for pandemic control (Yao et al. 2021).

Outdoor and indoor airborne SARS-CoV-2 surveillance under different conditions and contexts is important to provide scientific knowledge about the presence of SARS-CoV-2 RNA in aerosols with multiple possible uses such as designing practical screening strategies as community testing using air instead testing on an individual level. In a second step, the more studies showing success in culturing the virus from aerosol samples, the more evidence demonstrating the aerosol airborne transmission route of the virus, as it is mentioned in the report by Greenhalgh et al. (2021). Robust surveillance methods to test the environmental presence of SARS-CoV-2 would be very informative and useful from the point of view of public health to allow safe resumption of normal activities. The multiple methodological options developed in the different published and further studies would also provide knowledge about the feasibility of low versus intermediate and high-volume air samplers combined with the different RNA extraction kits designed for environmental samples to perform airborne SARS-CoV-2 surveillance tested by reverse transcriptase qPCR (RT-qPCR).

Although the public health sectors implemented several control strategies, such as social distancing, hygienic measures, and the development of anti-viral drugs and vaccines (Attia et al. 2021), the situation is still critical due to several challenges such as the clinical impacts of the emerged SARS-CoV-2 variants on the pathogenesis of the virus and vaccine efficacy (Shehata et al. 2021). According to the literature, COVID-19 infection risks are higher in healthcare workplaces than in nonhealthcare workplaces (Fawzy et al. 2021).

To contribute to the existing efforts in this knowledge, the aim of this study was to determine the presence of SARS-CoV-2 RNA in outdoor air samples from a relatively high PM contaminated area as well as in various hospital and non-hospital indoor settings naturally and mechanically ventilated.

## Methods

### Outdoor sampling surveillance

The area of study where the outdoor sampler was located (Santander Bay, Cantabria, northern Spain) has been described elsewhere (Arruti et al. 2010, 2011; Hernández-Pellón and Fernández-Olmo 2019a,b). Within this area, Maliaño was selected for two reasons: The first reason was that it is a town in Cantabria with more than 10,000 inhabitants that presented a higher incidence of COVID-19 in the first two waves. The second reason is the relatively high PM_10_ levels measured historically in this town; this led to the approval of a local air quality plan due to PM_10_ daily exceedances in 2012 (Fernández-Olmo et al. 2016). Five samples in Maliaño were collected between November and December 2020 (November 12th, 17th, and 25th and December 4th and 11th). Meteorological and air quality data during the sampling days were obtained from the Guarnizo station, located only 1.14 km from the sampling site (CIMA 2022). The temperature ranged between 14 ℃ and 18 ℃, except on December 4th (6.7 ℃); a relatively low wind speed was measured (below 3.2 m/s); sunny days were found in the sampling days of November while precipitation appeared in early December, which reduced the PM_10_ levels, from 18 to 19 μg/m^3^ in November to 4 and 11 μg/m^3^ measured on December 4th and 11th.

Following the published methodology for the detection of airborne viruses (Pan et al. 2019; Setti et al. 2020a; Linillos-Pradillo et al. 2021), outdoor aerosol daily samples were collected from a gravimetric air sampler (Dekati PM_10_ Impactor), with separation of coarse particles (PM_10-2.5_, i.e., with aerodynamic diameter < 10 μm and ≥ 2.5 μm) and fine particles (PM_2.5_, i.e., with an aerodynamic diameter < 2.5 μm) using a flowrate of 30 L/min (total sampling air volume of 43.2 m^3^); the sampler was placed on the rooftop of a public building of the town. Polycarbonate and Teflon (polytetrafluoroethylene, PTFE) filters were used to collect coarse and fine particles, respectively.

### Indoor sampling surveillance

Characteristics of indoor aerosol samples are described in detail in Tables 1, 2, and 3 and Supplementary Table S1. The sampling of aerosols in indoor environments was carried out by using personal PM samplers consisting of a personal pump (SKC Aircheck XR5000) with a flow rate of 3 L/min, connected to a particle impactor (SKC PMI coarse), where two Teflon (PTFE) filters are located, also allowing the separation of the coarse and fine fractions. In addition, the identification of the virus in the pre-filter (or impaction disc) was determined (> 10 µm). This personal impactor works in the same way as the one used in outdoor sampling, but takes advantage of its flexibility to be placed in different indoor environments and the relatively low noise of the air pump. Total air sampling volume ranged from 0.71 to 4.68 m^3^ (see Supplementary Table S1). Some additional non-segregated samples were also collected using a cassette containing a 37 mm PTFE filter of 0.3 μm pore size. The portable pump was placed in a bag and then on a hanger approximately 1.5 m above the ground (see Supplementary Figs. S1 and S2).

**Table 1:** Non-hospital indoor sampling surveillance results

*Filter code*	*Location*	*Air ventilation*	*Pre-filter (*> *10 µm)*	*Coarse fraction (2.5–10 µm)*	*Fine fraction (*< *2.5 µm)*
*EN01*	*University of Cantabria (UC) classroom (Nursing). First entry (lecturer)*	*Natural ventilation*	*neg*	*neg*	*neg*
*EN02*	*UC classroom (Nursing). Second entry*	*Natural ventilation*	*neg*	*neg*	*neg*
*EN03*	*UC classroom (Nursing). First entry (lecturer)*	*Natural ventilation*	*neg*	*neg*	*neg*
*EN04*	*UC classroom (Nursing). Second entry*	*Natural ventilation*	*neg*	*neg*	*neg*
*ME01*	*UC classroom (Medicine). Right entry*	*Mechanical ventilation*	*neg*	*neg*	*neg*
*ME02*	*UC classroom (Medicine). Left entry*	*Mechanical ventilation*	*neg*	*neg*	*neg*
*ME03*	*UC classroom (Medicine). Right entry*	*Mechanical ventilation*	*neg*	*neg*	*neg*
*ME04*	*UC classroom (Medicine). Left entry*	*Mechanical ventilation*	*neg*	*neg*	*neg*
*BC01*	*UC. Central library-Paraninfo. Study room (central zone)*	*Mechanical ventilation*	*neg*	*neg*	*neg*
*BC02*	*UC. Central library-Paraninfo. Dining room*	*Mechanical ventilation*	*neg*	*neg*	*neg*
*BC03*	*UC. Central library-Paraninfo. Study room (entrance)*	*Mechanical ventilation*	*neg*	*neg*	*neg*
*BC04*	*UC. Central library-Paraninfo. Study room (central zone)*	*Mechanical ventilation*	*neg*	*neg*	*neg*
*BC05*	*UC. Central library-Paraninfo. Dining room*	*Mechanical ventilation*	*neg*	*neg*	*neg*
*BC06*	*UC. Central library-Paraninfo. Study room (entrance)*	*Mechanical ventilation*	*neg*	*neg*	*neg*

**Table 2:** Hospital indoor sampling surveillance results in the pediatric nasopharyngeal testing room at Liencres Hospital

*Filter code*	*Location (distance from source, meters)*	*Air ventilation*	*Observations*	*Pre-filter (*> *10 µm)*	*Coarse fraction (2.5–10 µm)*	*Fine fraction (*< *2.5 µm)*
*LI01*	*Pediatric nasopharyngeal testing room (*< *1 m)*	*Natural ventilation*	*9 positive cases out of 124 (7.3%)*	*neg*	*neg*	*neg*
*LI02*	*Pediatric nasopharyngeal testing room (3 m)*	*Natural ventilation*	*9 positive cases out of 124 (7.3%)*	*neg*	*neg*	*neg*
*LI03*	*Pediatric nasopharyngeal testing room (*< *1 m)*	*Natural ventilation*	*14 positive cases out of 143 (9.8%)*	*neg*	*neg*	*neg*
*LI04*	*Pediatric nasopharyngeal testing room (3 m)*	*Natural ventilation*	*14 positive cases out of 143 (9.8%)*	*neg*	*neg*	*neg*
*LI05*	*Pediatric nasopharyngeal testing room (*< *1 m)*	*Natural ventilation*	*11 positive cases out of 240 (4.6%)*	*neg*	*neg*	*neg*
*LI06*	*Pediatric nasopharyngeal testing room (3 m)*	*Natural ventilation*	*11 positive cases out of 240 (4.6%)*	*neg*	*neg*	*neg*
*LI07*	*Pediatric nasopharyngeal testing room (*< *1 m)*	*Natural ventilation*	*7 positive cases out of 189 (3.7%)*	*neg*	*neg*	*neg*
*LI08*	*Pediatric nasopharyngeal testing room (3 m)*	*Natural ventilation*	*7 positive cases out of 189 (3.7%)*	*neg*	*neg*	*neg*

**Table 3:** Hospital indoor sampling surveillance results at HUMV Hospital

*Filter code*	*Location*	*Air ventilation*	*Observations*	*Pre-filter (*> *10 µm)*	*Coarse fraction (2.5–10 µm)*	*Fine fraction (*< *2.5 µm)*
*HV01*	*HUMV COVID plant, “Hallway,” Transit area*	*Mechanical*		*neg*	*neg*	*neg*
*HV02*	*HUMV COVID plant, “Dirty zone,” COVID plant, Protective equipment removal area*	*Mechanical*		*neg*	*neg*	*neg*
*HV03*	*HUMV COVID plant, single occupancy room*	*Mechanical*	*TSP, non-size-segregated sample*	neg*		
*HV04*	*HUMV COVID plant, single occupancy room*	*Mechanical*	*Only 8 out of 25 h with the patient in the room, then moved to ICU*	*neg*	*neg*	*neg*
*HV05*	*HUMV COVID plant, single occupancy room*	*Mechanical*	*Patient with cough; stays for 26 h in room*	*neg*	*neg*	*positive*
*HV06*	*HUMV COVID plant, single occupancy room*	*Mechanical. Negative pressure high-flow room*	*TSP, non-size-segregated sample*	neg*		
*HV07*	*HUMV COVID plant, single occupancy room*	*Mechanical*	*Long-stay patient, probably non-transmissible at sampling*	*neg*	*neg*	*neg*
*HV08*	*HUMV COVID plant, single occupancy room*	*Mechanical*	*Long-stay patient, probably non-transmissible at sampling*	*neg*	*neg*	*neg*

*A cassette with a PTFE filter was used to collect total suspended particles (TSP) without fractionation

*neg*, N1 and N2 genes negative RT-qPCR results; *positive*, both N1 and N2 genes positive

#### Non-hospital indoor sampling surveillance

Regarding indoor air sampling at non-hospital wards, 14 aerosol samples were collected between April and June 2021 (total air volume ranged from 0.71 to 1.77 m^3^), after the relevant permits were obtained for aerosol sampling at two different classrooms of the University of Cantabria (UC): the first-year classroom of the Degree in Nursing (Faculty of Nursing), and the first-year classroom of the Degree in Medicine placed in the main conference room of the Faculty of Medicine; and two areas of the central library-Paraninfo of the UC: study room and dining room. Supplementary Figs. S1 and S2 show photographs of their location in the Faculty of Nursing and the Central Library of the University of Cantabria as an example, respectively.

The first-year classroom of the Degree in Nursing is a large classroom with a capacity of 90 students spaced 1.5 m distance from each other. It is a naturally ventilated open ward where per protocol: For each hour of lecture, 50 min, the windows are partially opened (in tilt-and-turn window position), and for the rest of 10 min, all windows are fully opened.

The first-year classroom of the Degree in Medicine and the study room and the dining room of the University library have mechanical ventilation with air renovation. The university library has a new air renovation equipment consisting of M5 + F7 filtration (according to EN779:2012) of outdoor air with a volumetric air flow rate of 45 m^3^/h/person in the study room and 28.8 m^3^/h/person in the dining room, corresponding to 2.51 and 3.23 air exchanges/h, respectively. The classroom of the Degree of Medicine has an old air renovation equipment, and no data on air renovation was available. Both students and lecturers wore a mask during the lessons and when staying at the library except in the dining room when eating.

#### Hospital indoor sampling surveillance

##### Pediatric nasopharyngeal testing room at Liencres Hospital

This room is next to one of the hospital entrances, and it is a naturally ventilated open ward, where children < 10 years old were cited because of a suspect of symptoms of COVID-19, or in case of fulfilling the non-symptomatic close contact definition in a contact tracing context. In this room, a range of children between 124 and 240 per day entered for less than a minute, wearing masks that were taken off only when swabbing. Windows were partially opened in tilt-and-turn position all continuously during all working days. Liencres Hospital is a small hospital where the epidemiological unit responsible for all contact tracing at the provincial level was based. This hospital does not have a hospital emergency care service and does not treat infectious patients, so it can be considered free of COVID-19 patients. Eight samples (two samples per day) were collected between January and February 2021 (14, 21, and 28 January and 4 February) by the same personal samplers used at the indoor university sampling campaign; total air volume ranged from 1.48 to 1.79 m^3^. Positivity rates of children in their nasopharyngeal testing on these four days of aerosol sampling were in the range of 3.7% and 7.3% (see Table 2).

##### Clinical areas of COVID plant at HUMV Hospital

HUMV Hospital is a tertiary hospital where the main COVID-19 hospitalization in the province was centered, creating a specific COVID plant for these patients. In this plant, aerosol samples were collected on 19 January 2021 in two different spaces: a hallway in the context of a transit area and a “dirty area” where healthcare workers take off their personal protective equipment (PPE). The total air sampled volume was 4.32 m^3^.

##### Occupancy rooms of COVID plant at HUMV Hospital

Rooms for patients hospitalized on the COVID floor were single occupancy rooms, with closed door and dedicated bathroom. Personal samplers were placed in six different rooms on 19 and 26 January and 25 May 2021 (total air volume ranged from 1.44 to 4.68 m^3^). In every room, the sampler was situated next to the patient’s bed (1.5 m). In one of the rooms, the patient was transferred to the ICU (during the sampling, the patient remained in the room for 8 h). In the rest of the samples, the COVID + patients remained in their rooms all sampling time long. Moreover, one of the rooms was a high-flow ward with negative pressure and increased air renovation. The rest of the rooms were non-negative pressure mechanically ventilated (see Table 3).

The project was approved by the clinical ethics committee of Cantabria (CEIC), internal code: 2020.401, and the ethics committee of the UC (CEUC). In addition, informed consent was obtained from patients in rooms on the COVID-19 plant when sampling at HUMV.

### RNA extraction and analysis.

#### RNA extraction protocol. Sample processing steps

Upon completion of each sampling, the bags containing the impactors were immediately sent to the laboratory for processing as soon as they were received. The entire filter handling process was carried out in a laminar flow hood under sterile conditions to avoid cross-contamination. All instruments used were sterilized, and with the exception of the tweezers used to pick up the filters, the rest of the material was discarded after use in biological waste containers. Specifically, filters were taken and folded with sterile tweezers into 5 mL Eppendorf tubes. RNA extraction was carried out with TRIzol® reagent (ThermoFisher Scientific, Waltham, Massachusetts, USA), which is a single-phase phenol-guanidine isothiocyanate reagent designed to isolate and separate RNA, DNA, and protein fractions. Then, 1 mL of Trizol was poured into the 5 mL tubes. The filters were then thoroughly vortexed. It was ensured that any possible particles with biological material were embedded in the Trizol mixture. This mixture was frozen at − 80℃ for further processing together with other sample collection batches. The frozen samples were defrosted on ice, and the RNA isolation process was continued cold (on ice). Filters were removed, ensuring that they did not drag Trizol with them. To each 1 mL of Trizol, 200 µL of chloroform was added to isolate RNA in an aqueous phase. After centrifugation (all centrifugations were at 13,000 rpm and 4 degrees), the aqueous phase was taken to precipitate RNA with isopropanol (volume 1:1). The RNA was washed with 75% ethanol and finally resuspended in sterile RNAase-free water.

#### RT-qPCR assays

Once the RNA was isolated in a total volume of 15 µL, 2 µL were used for the PCR. Specifically, two genes of the SARS-CoV-2 were analyzed (N1 and N2) twice per sample. RT-PCR (reverse transcription and amplification of the DNA formed) was performed in a single step. Takara (TAKARA BIO INC., Kusatsu, Japan) kit [RR064A (One Step PrimeScript™ RT-PCR Kit (Perfect Real Time)] and the IDT (IDT, Coralville, Iowa, USA) probes [SARS-CoV-2 (2019 nCoV) CDC RUO Primers and Probes] were used. The reaction mixture was as indicated in Supplementary Table S2.

**Fig. 1:**
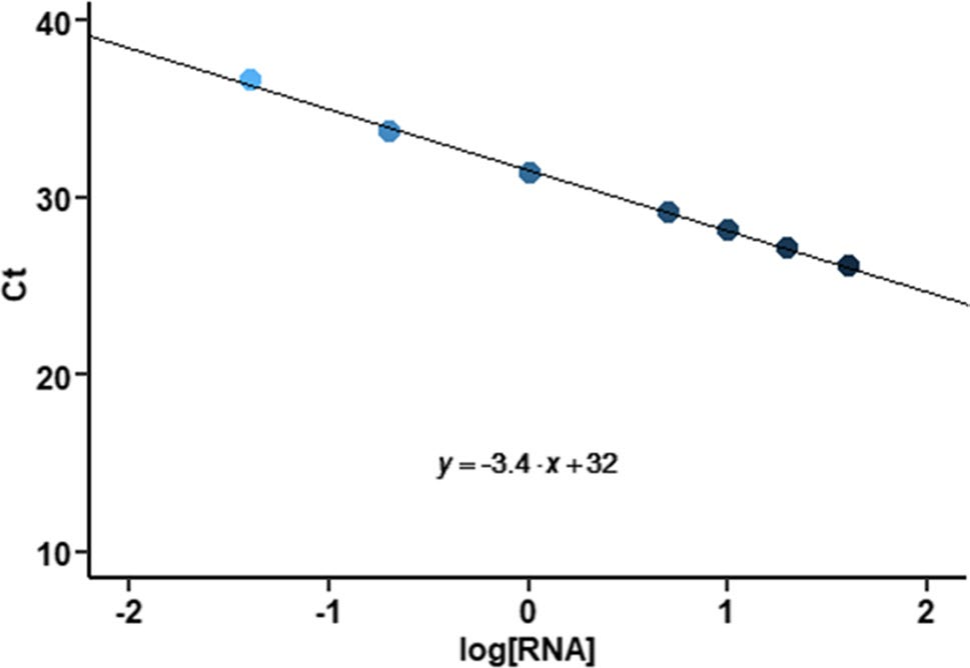
Sensitivity of the SARS-CoV-2 RNA detection technique. RNA concentration units, ng/μL

The RT-PCR program was run on the Applied Biosystems 7500 Real-time PCR System (ThermoFisher Scientific, Waltham, Massachusetts, USA). The program consists of two distinct parts. The first part (reverse transcription, RT) consisted of a step at 42 ℃ for 5 min, followed by a 10-s inactivation step at 95 ℃. In the second part (amplification, PCR), 40 cycles of two steps were performed, 5 s at 95 ℃ followed by 34 s at 60 ℃. All reactions were performed with both positive and negative controls. Non-viable SARS-CoV-2 RNA was used as positive controls. These control tests were successfully accomplished. Furthermore, a concentration curve was first performed with different RNA ratios to determine the sensitivity of the technique. Hence, the non-viable SARS-CoV-2 RNA, used for the positive PCR controls, has also been used to create a concentration curve starting from the original concentration (40 ng/µL). The following serial dilutions were generated for the PCR: 40 ng/µL, 20 ng/µL, 10 ng/µL, 5 ng/µL, 1 ng/µL, 0.2 ng/µL, 0.04 ng/µL, and 0.008 ng/µL (see Fig. 1). Positive samples were identified as those with a cycle threshold (C_t_) cutoff of 40.0. According to Fig. 1, we were able to detect 0.04 ng/µL as the lowest concentration of RNA. Calibration points, control tests, and samples were measured in duplicate, reaching a precision of the PCR readings of 0.63% for the positive controls and calibration points and 5.06% for the one sample that tested positive.

In addition to the negative and positive controls used when samples were analyzed, the whole experimental methodology was checked by doping laboratory blank filters. As for the concentration curve analysis, non-viable SARS-CoV-2 RNA was used to dope the blank filters at different concentrations. Thus, an RNA isolation with Trizol was performed with the previously doped filters. Results showed that only those laboratory blank filters doped with non-viable SARS-CoV-2 RNA were amplified after the PCR.

## Results

Firstly, samples collected in outdoor air (Maliaño) were analyzed, all of them being negative in both coarse and fine fractions. With respect to indoor sampling, Tables 1, 2, and 3 show the results from the non-hospital indoor sampling campaign, the pediatric nasopharyngeal testing room at Liencres Hospital, and the indoor surveillance campaign in HUMV Hospital, respectively. As can be seen in Table 3, only sample HV05 was positive for both N1 and N2 genes (in particular the fine fraction), collected in one occupancy room of the COVID plant at HUMV Hospital, where a patient with positive PCR and cough was present. This sample corresponded to one of the largest air volume collected, as shown in Supplementary Table S1 (4.68 m^3^). In this RT-PCR, the mean C_t_ value was 28, which, according to Fig. 1, corresponded to a log[RNA] mean of 1.17 (i.e., 15 ng/µL). The rest of the samples were negative, both in the fine and coarse fractions, as well as in the pre-filter, which collected particles coarser than 10 µm.

## Discussion

In terms of outdoor surveillance, no presence of SARS-CoV-2 was observed in our study conducted in Maliaño, using polycarbonate and Teflon (PTFE) filters for PM_10-2.5_ and PM_2.5_ fractions. Samples were collected between November and December 2020, a period with sustained community transmission, which corresponded to a decreasing trend of the third wave in Cantabria: 193, 176, 107, 98 and 61 daily cases on November 12th, 17th, and 25th and December 4th and 11th, respectively (CNE 2022). The highest PM_10_ daily levels were measured on November 12th and 17th (18 μg/m^3^ and 19 μg/m^3^), when higher pressure, lower wind speed, and the absence of rain were recorded; however, although these days also presented a higher daily incidence, these samples were negative. Subsequently, the incidence was reduced, and the meteorological conditions recorded during early December favored lower PM_10_ concentration levels, 4 μg/m^3^ and 11 μg/m^3^ measured on December 4th and 11th, where rainfall was 22.5 mm and 5.3 mm, respectively.

Our results have to be interpreted in a context of a relatively low-volume gravimetric outdoor air sampling (30 L/min for 24 h, i.e., 1.8 m^3^/h) as well as Setti et al. (2020a) results (38.3 L/min for 24 h). However, our results did not support Setti et al.’s results in the industrial area of Bergamo (Italy), where SARS-CoV-2 RNA was detected under conditions of atmospheric stability and high PM concentrations in outdoor PM_10_, over a continuous 3-week sampling period, from February 21st to March 13th, 2020, even though our samples were also placed in a highly industrialized area (Arruti et al. 2010, 2011; Fernández-Olmo et al. 2016; Hernández-Pellón and Fernández-Olmo 2019a,b). The Setti et al. epidemiological context probably encompasses a more severe COVID-19 burden as the Bergamo area was the epicenter of the Italian COVID-19 epidemic. Another explanation may be that our sampler was placed on the rooftop of a building (several floors high) under the hypothesis that at that altitude SARS-CoV-2 RNA is not already present in aerosols or it is present in quantity below the threshold necessary to be detected. It is also plausible that atmospheric and meteorological situations could have also affected the dispersion capacity of the atmosphere and the state of pollution concentration level. Promoted by the positive results of Setti et al., further studies were ongoing in Milan and Naples (Italy), Madrid and Barcelona (Spain), Bruxelles (Belgium), and New York – under the RESCOP (Research group on COVID-19 and Particulate Matter) International Research Initiative. Up to our knowledge, only Madrid results have been published (Linillos-Pradillo et al. 2021), supporting our results since no presence of SARS-CoV-2 during the month of May 2020 in PM_10_, PM_2.5_, and PM_1_ (particles with aerodynamic diameter < 1 μm) filters collected outdoor by a high-volume sampler (flowrate of 30 m^3^/h for 17.5 to 24 h) was observed, even though Madrid was the epicenter of the Spanish epidemic at the time of sampling. For the moment, a significant gap exists in the role of ambient air pollution in the spread of SARS-CoV-2. On the other hand, it is better known that air pollution is associated with an increase in host susceptibility to viral infections including SARS-CoV-2, and that also worsens the severity of viral infections including COVID-19, probably mediated by the increase of the risk of cardiovascular complications, chronic obstructive pulmonary disease (COPD), among other conditions (Domingo et al. 2020); so, environmental policies for the reduction of pollution levels in terms of PM would therefore be equally appropriate. In addition to the RESCOP initiative, other outdoor sampling studies have been recently published; thus, while Chirizzi et al. (2021) found negative results for the presence of SARS-CoV-2 in northern (Veneto) and southern (Apulia) regions of Italy, Kayalar et al. (2021) reported a 10% of positive samples for PM_10-2.5_, PM_2.5_, and > 10 μm fractions collected from 13 sites including urban and urban-background locations and hospital gardens in 10 cities across Turkey between 13th of May and 14th of June 2020 by using both low and high-volume samplers, with a total of 203 daily samples. The highest percentages of detection were from hospital gardens and in the PM_2.5_ fraction, suggesting that SARS-CoV-2 would be airborne present, especially at sites close to the infection hot-spots (Kayalar et al. 2021).

Regarding our indoor sampling surveillance, only one positive RT-qPCR sample was obtained, corresponding to a patient room in the context of an indoor Non-ICU patient hospital environment. The standardization and validation of methods and processes for SARS CoV-2 environmental sampling is necessary in order to obtain consistent and comparable results (Robotto et al. 2021). However, the need for immediate results on the possible presence of SARS-CoV-2 RNA in aerosol samples has led to a context of lack of comparability in many published research works, leading to contradictory results with respect to the positivity rates of SARS-CoV-2 in indoor samples, as summarized in recent published reviews (Birgand et al. 2020; Borges et al. 2021; Comber et al. 2021; Noorimotlagh et al. 2021; Dinoi et al. 2022). Several reasons may explain these contradictory results and particularly our limited positive results in indoor hospital settings compared to the results from other studies: air sampling method and total air sampling volume, air renovation and viral load, distance between sampling site and patient, any possible degradation during RNA extraction, and RT-qPCR assay limitations.

Considering the total air sampling volume (i.e., the product of the volumetric flowrate and the sampling duration), some recent studies have found higher positivity rates when the total volume of air sampled is increased. Thus, Ang et al. (2021) reported no positive detection of airborne SARS-CoV-2 (0/3 positive samples in negative pressure isolation rooms) when 24 m^3^ of air was sampled (flowrate of 50 L/min) in a hospital airborne SARS-CoV-2 surveillance in Singapore, whereas upon increasing the total air volume to 72 m^3^ (flowrate of 150 L/min), the positive rate in detecting the presence of SARS-CoV-2 increased to 60–87.5%. Dubey et al. (2021) also found an increase in the positivity rate in an Indian hospital from 28.6 to 54.8% when the total air volume increased from 0.09 to 1.6 m^3^. Other previous studies support the relationship between sampling flowrate and airborne SARS-CoV-2 detection. Guo et al. (2020) and Ding et al. (2021) have only a positive SARS-CoV-2 detection when using 300 L/min and 500 L/min sampler, respectively. However, the sampling duration in these two studies was very short, 30 min in Guo et al. (2020) and 2 min in Ding et al. (2021). The high flow rates used in these two studies appear not to damage viral RNA upon impact. Finally, all samples collected in non-healthcare indoor settings in three Italian communities using different flowrates (6 to 28 m^3^ of sampled air) were negative (Conte et al. 2022).

Regarding results from hospital studies with lower flowrate air samplers, and therefore lower total sampling air volume, positive results have been reported by Chia et al. (2020) using 5–9 L/min air samplers, Santarpia et al. (2020) by using a mobile personal sampler with 4 L/min air flowrate, or Grimalt et al. (2022), using a similar flowrate for 4 h. However, airborne SARS-CoV-2 concentrations were significantly higher in these studies (thousands of copies/m^3^ of air) compared with the rest of the studies with positive detection with high-flowrate samplers (normally with tens to hundreds of copies/m^3^ of air) (Guo et al. 2020; Liu et al. 2020; Ang et al. 2021; Ding et al. 2021). In this respect, in a systematic review including 24 cross-sectional observational studies published up to October 2020, the median SARS-CoV-2 RNA concentrations were 1.0 × 10^3^ copies/m^3^ in clinical areas and 9.7 × 10^3^ copies/m^3^ in the air of toilets or bathrooms (Birgand et al. 2020). It is remarkable that in this systematic review, 11 out of the 19 studies with hospital samples in non-ICU patient environments and 5 out of 8 with samples in staff areas showed no successful detection of airborne SARS-CoV-2.

Concerning air ventilation conditions, the level of air renovation in the patient rooms on the COVID ward can be considered as high, although not all rooms had the same characteristics (only one room had negative pressure). On the other hand, it is plausible that viral load and the corresponding positive detection rate in aerosols would be associated with the distance between the sampling site and the patient; the furthest distance, the lesser the positive detection rate (Guo et al. 2020; Ang et al. 2021). For example, all the air samples (*n* = 33) collected at 2–5 m away from COVID-19 patients’ beds at an Iranian hospital were negative (Vosoughi et al. 2021).

Different results among published studies can also be explained by differences in analytical approaches. Our qPCR assay targeted nucleocapsid protein gene (N1 and N2 genes). Some authors have reported that the envelope protein gene (E-gene) would be more sensitive than N-genes both in terms of positive detection rate and C_t_ values (Ang et al. 2021). Other authors also incorporate NA-dependent RNA polymerase (RdRP) genes (Kayalar et al. 2021; Linillos-Pradillo et al. 2021). Nevertheless, the positive control of our qPCR assays and the fact that our amplification approach can be considered similar to the protocol developed by Corman et al. (2020) published on the WHO website (Corman et al. 2020) seem to support that our extraction and assay efficiency be enough to achieve detection in potential SARS-CoV-2 RNA samples.

Our university indoor classroom context was a highly exterior ventilated context at the Nursing Faculty and a mechanical ventilation context at the Faculty of Medicine, where air samplers were placed at a certain distance from lecturers and from the majority of the students (> 3 m). None of the students present in the classrooms and in the central library were positive at the time of sampling up to our knowledge. Due to the preventive measures for face-to-face teaching, including the use of masks, and monitoring and isolation of cases and close contacts, it is highly improbable the presence of SARS-CoV-2 during sampling, minimizing the possibility that our negative results were false negatives results. The central library-Paraninfo of the UC lacks natural ventilation but has new air recirculation equipment; study room and dining room air samplers were also placed at a certain distance from students and preventive measures were similar for students including the use of masks. Thus, students who were present in these indoor spaces wore masks at all times, which would imply that if they were SARS-CoV-2 transmissible, its level in exhaled air would be significantly lower, up to 48% and 77% lower in fine and coarse aerosols, respectively, according to Adenaiye et al. (2021). Only those students eating in the Central Library dining room (samples BC2 and BC5) did not wear masks.

Lastly, in relation to the pediatric nasopharyngeal testing room at Liencres Hospital, this indoor context was also an exterior ventilated context, in which one air sampler was closed to the children (< 1 m) and the other was at a greater distance from the children (> 3 m). During the nasal swabbing, the children took off their masks and a number of children (range 3.7–7.3%) were positive for their PCRs being transmissible at the time of sampling, so the possibility of false negatives results cannot be discharged for the above-mentioned reasons such as the low sampling volume. However, the absence of infections among the nurses in attendance to take their samples (during the sample time and all over the epidemic until now) supports the existence of safe non-transmissibility conditions in terms of occupational health.

## Conclusions

In terms of outdoor surveillance, no presence of SARS-CoV-2 for PM_10-2.5_ and PM_2.5_ samples was observed in our study conducted in northern Spain in a relatively high PM_10_-contaminated area during November and December 2020 with sustained community transmission. In terms of our indoor surveillance using low-flowrate air samplers (3 L/min) and low sampling air volume (0.71–4.68 m^3^), no presence of SARS-CoV-2 was either observed in two classrooms during lectures at university, in the central library-Paraninfo of the UC at Santander, Spain. Regarding our hospital indoor surveillance using the same air samplers, SARS-CoV-2 was not detected in aerosols in the pediatric nasopharyngeal testing room at Liencres Hospital or in the clinical areas of the main hospital of the province (HUMV). SARS-CoV-2 was positively detected only in one patient room. Our indoor results support the maintenance of preventive measures such as high air ventilation conditions, use of masks, and social distance.

## Supplementary Information

Below is the link to the electronic supplementary material.

Supplementary file1 (DOCX 521 KB)

## Data Availability

Not applicable.
