# Supplementary material for: SARS-CoV-2 surveillance in indoor and outdoor size-segregated aerosol samples
Source: Environ Sci Pollut Res Int. 2022 Apr 21;29(42):62973–83. doi: 10.1007/s11356-022-20237-7 (PMC9023038; doi:10.1007/s11356-022-20237-7)
Supplement: Supplementary file 1 — Supplementary file1 (DOCX 521 KB) [file 11356_2022_20237_MOESM1_ESM.docx]

**SUPPLEMENTARY MATERIAL**

SARS-CoV-2 surveillance in indoor and outdoor size-segregated aerosol samples

**Álvaro del Real ^1^, Andrea Expósito ^2^, Laura Ruiz-Azcona ^3^, Miguel Santibáñez ^3,4^, and Ignacio Fernández-Olmo ^2*^.**

^1^ Medicine and Psychiatry Department, University of Cantabria, Av. Cardenal Herrera Oria, 2, 39011 Santander, Spain

^2^ Departamento de Ingenierías Química y Biomolecular, Universidad de Cantabria, Avda. Los Castros s/n, 39005 Santander, Cantabria, Spain

^3^ Global Health Research Group. Dpto Enfermería, Universidad de Cantabria, Avda. Valdecilla, s/n. 39008 Santander, Cantabria, Spain

^4^ Nursing Research Group, IDIVAL, Calle Cardenal Herrera Oria s/n, 39011 Santander, Cantabria.

*Corresponding author: Ignacio Fernández Olmo. Departamento de Ingenierías Química y Biomolecular, Universidad de Cantabria, Avda. Los Castros s/n, 39005, Cantabria, Spain. Phone: +34 942 206745. E-mail address: fernandi@unican.es

Ignacio Fernández-Olmo and Miguel Santibáñez share senior authorship.

***SUPPLEMENTARY TABLES***

***Table S1.*** *Characteristics of indoor aerosol sampling*

| ***Filter code*** | ***Location (distance from source, meters)*** | ***Date***  ***Day/month/year*** | ***Duration (min)*** | ***Air volume (m^3^)*** |
| --- | --- | --- | --- | --- |
|  | ***Non-hospital indoor surveillance*** |  |  |  |
| *EN01* | *University of Cantabria (UC) Classroom (Nursing). First Entry (lecturer)* | *27/04/2021* | *488* | *1.46* |
| *EN02* | *UC Classroom (Nursing). Second Entry.* | *27/04/2021* | *478* | *1.43* |
| *EN03* | *UC Classroom (Nursing). First Entry (lecturer)* | *29/04/2021* | *540* | *1.62* |
| *EN04* | *UC Classroom (Nursing). Second Entry.* | *29/04/2021* | *542* | *1.63* |
| *ME01* | *UC Classroom (Medicine). Right Entry* | *04/05/2021* | *237* | *0.71* |
| *ME02* | *UC Classroom (Medicine). Left Entry* | *04/05/2021* | *240* | *0.72* |
| *ME03* | *UC Classroom (Medicine). Right Entry* | *07/05/2021* | *306* | *0.92* |
| *ME04* | *UC Classroom (Medicine). Left Entry* | *07/05/2021* | *305* | *0.92* |
| *BC01* | *UC. Central library-Paraninfo. Study room (central zone)* | *01/06/2021* | *568* | *1.70* |
| *BC02* | *UC. Central library-Paraninfo. Dining room* | *01/06/2021* | *579* | *1.74* |
| *BC03* | *UC. Central library-Paraninfo. Study room (entrance)* | *01/06/2021* | *572* | *1.72* |
| *BC04* | *UC. Central library-Paraninfo. Study room (central zone)* | *03/06/2021* | *590* | *1.77* |
| *BC05* | *UC. Central library-Paraninfo. Dining room* | *03/06/2021* | *589* | *1.77* |
| *BC06* | *UC. Central library-Paraninfo. Study room (entrance)* | *03/06/2021* | *583* | *1.75* |

***Table S1.*** *Characteristics of indoor aerosol sampling (continued)*

| ***Filter code*** | ***Location (distance from source, meters)*** | ***Date***  ***Day/month/year*** | ***Duration (min)*** | ***Air volume (m^3^)*** |
| --- | --- | --- | --- | --- |
|  | ***Hospital indoor surveillance.*** |  |  |  |
|  | ***Clinical area. Liencres Hospital.*** |  |  |  |
| *LI01* | *Paediatric nasopharyngeal testing room (<1 meter).* | *14/01/2021* | *493* | *1.48* |
| *LI02* | *Paediatric nasopharyngeal testing room (3 meters).* | *14/01/2021* | *493* | *1.48* |
| *LI03* | *Paediatric nasopharyngeal testing room (<1 meter)* | *21/01/2021* | *495* | *1.49* |
| *LI04* | *Paediatric nasopharyngeal testing room (3 meters).* | *21/01/2021* | *495* | *1.49* |
| *LI05* | *Paediatric nasopharyngeal testing room (<1 meter)* | *28/01/2021* | *598* | *1.79* |
| *LI06* | *Paediatric nasopharyngeal testing room (3 meters).* | *28/01/2021* | *598* | *1.79* |
| *LI07* | *Paediatric nasopharyngeal testing room (<1 meter)* | *04/02/2021* | *591* | *1.77* |
| *LI08* | *Paediatric nasopharyngeal testing room (3 meters).* | *04/02/2021* | *591* | *1.77* |
|  | ***Clinical areas. HUMV COVID plant*** |  |  |  |
| *HV01* | *HUMV COVID plant, “Hallway”, Transit area* | *19/01/2021* | *1440* | *4.32* |
| *HV02* | *HUMV COVID plant, “Dirty zone”, COVID plant, Protective equipment removal area* | *19/01/2021* | *1440* | *4.32* |
|  | ***HUMV COVID plant*** |  |  |  |
| *HV03* | *HUMV COVID plant, single occupancy room (<1.5 meters).* | *19/01/2021* | *480* | *1.44* |
| *HV04* | *HUMV COVID plant, single occupancy room (<1.5 meters).* | *26/01/2021* | *1560* | *4.68* |
| *HV05* | *HUMV COVID plant, single occupancy room (<1.5 meters).* | *26/01/2021* | *1560* | *4.68* |
| *HV06* | *HUMV COVID plant, single occupancy room (<1.5 meters).* | *26/01/2021* | *1080* | *3.24* |
| *HV07* | *HUMV COVID plant, single occupancy room (<1.5 meters).* | *25/05/2021* | *1116* | *3.35* |
| *HV08* | *HUMV COVID plant, single occupancy room (<1.5 meters).* | *25/05/2021* | *1116* | *3.35* |

***Table S2.*** *Composition of the reaction mixture to be added to each sample.*

| **Reactives** | **Mixture (µL) 1x** |
| --- | --- |
| Buffer | 10 |
| Takara ExTaq HS | 0.4 |
| RT Enzyme | 0.4 |
| Probe (Gen) | 1.5 |
| H_2_O | 5.7 |

*SUPPLEMENTARY FIGURES*


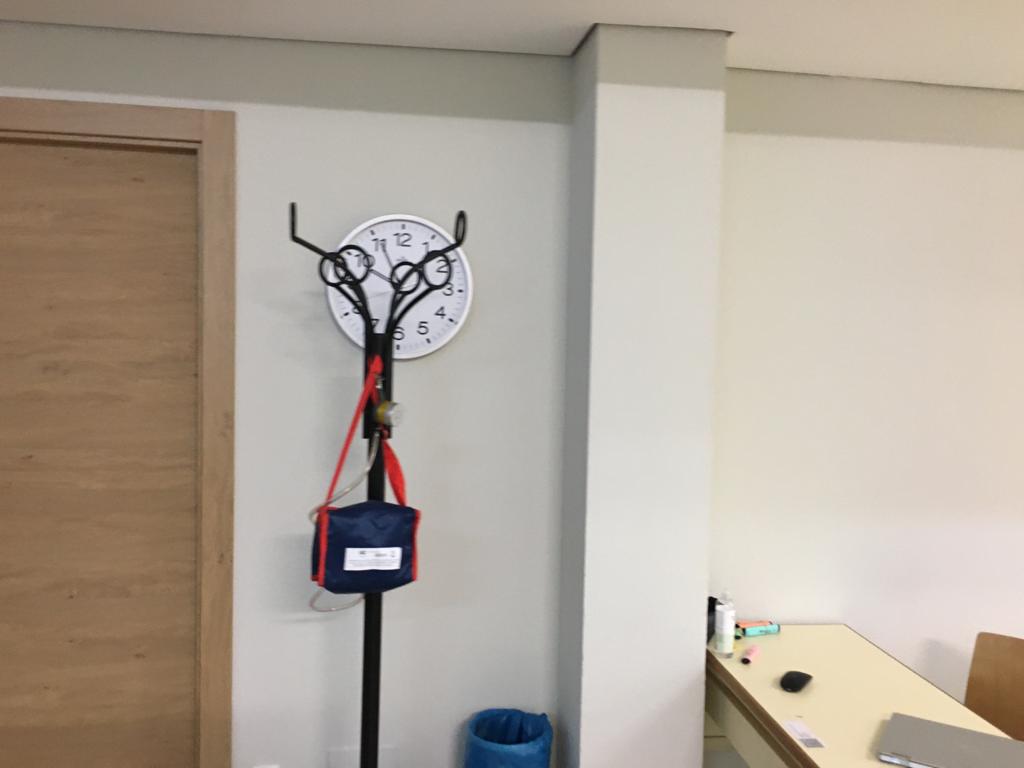


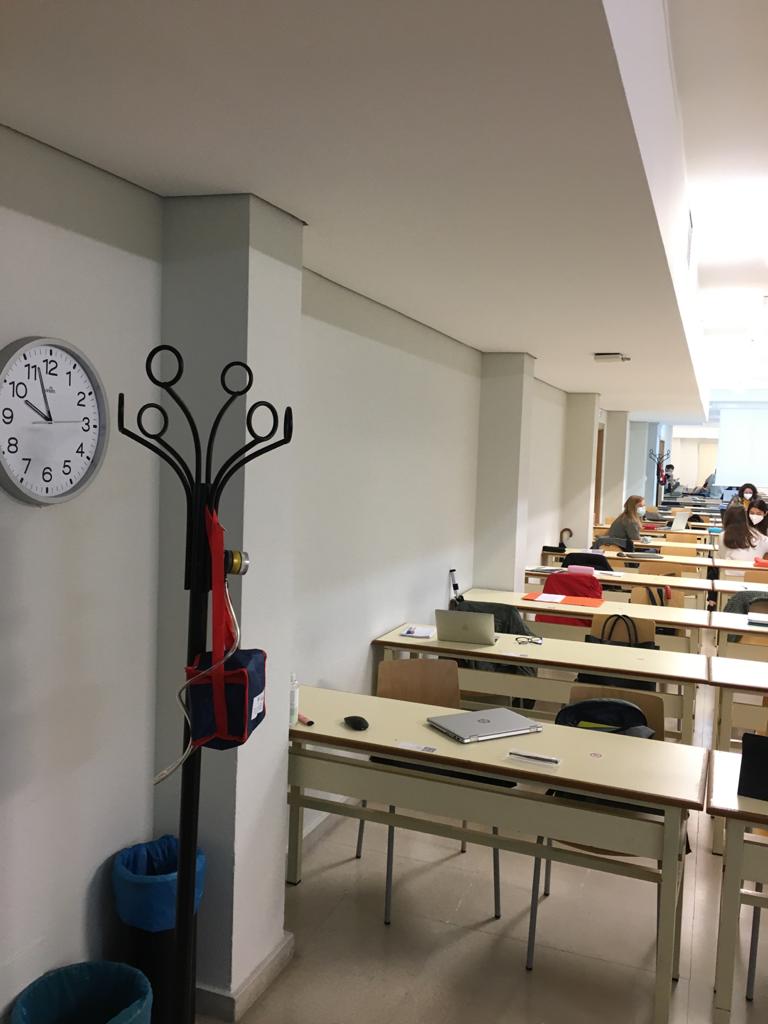


Figure S1. Aerosol sampling at the Nursing Faculty of the University of Cantabria (UC)


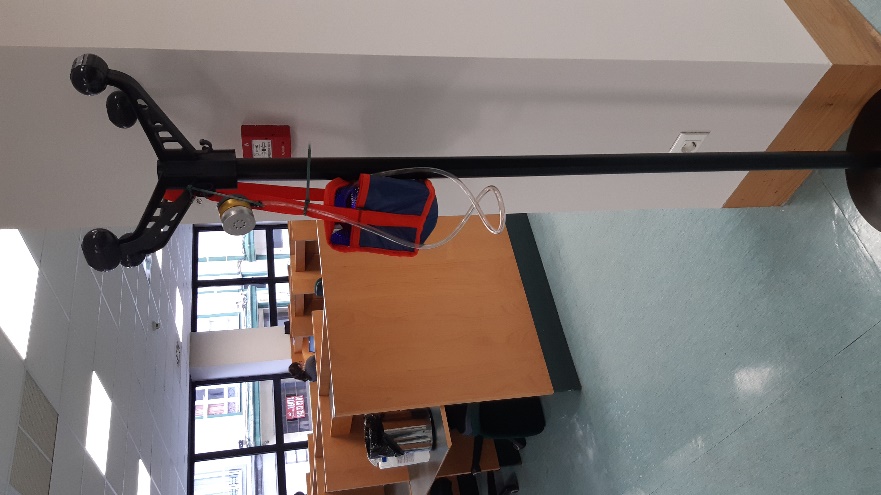


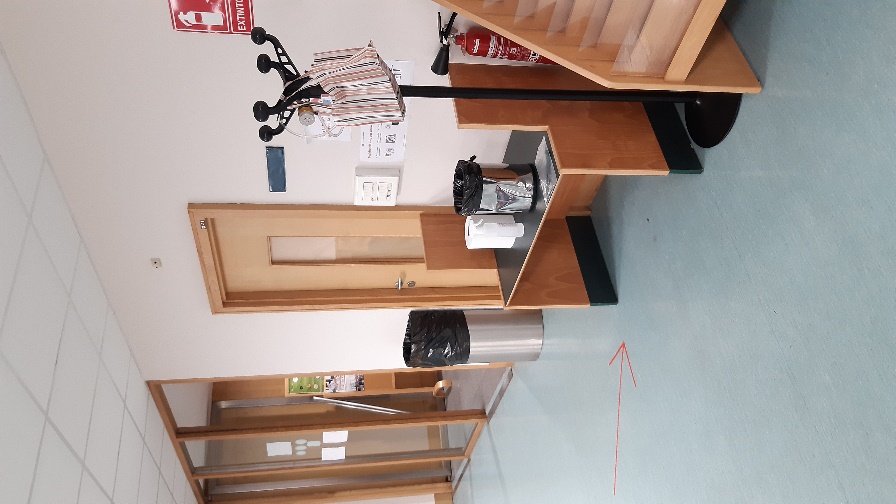

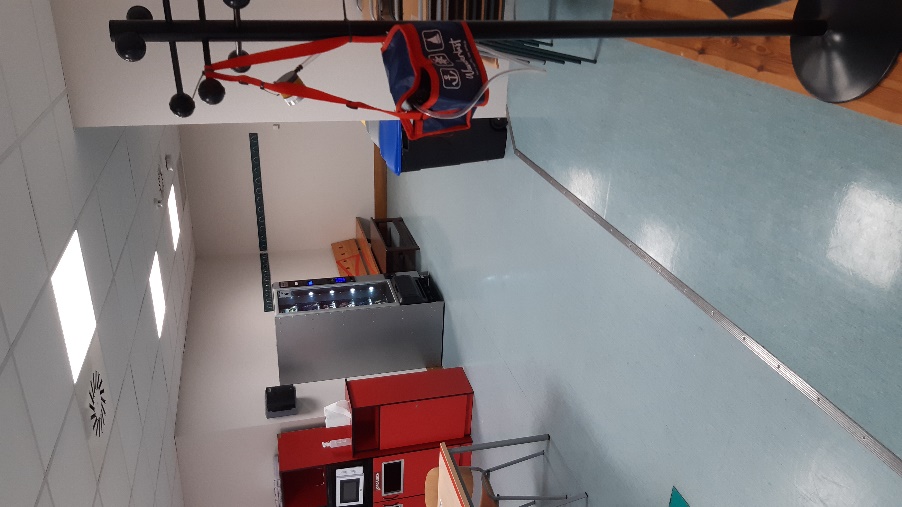


Figure S2. Aerosol sampling at the study and dining rooms of the library of the University of Cantabria (UC)
